# Growth behavior of titanium dioxide thin films at different precursor temperatures

**DOI:** 10.1186/1556-276X-7-89

**Published:** 2012-01-26

**Authors:** Sang-Hun Nam, Sang-Jin Cho, Jin-Hyo Boo

**Affiliations:** 1 Department of Chemistry and Institute of Basic Science, Sungkyunkwan University, Suwon, 440-746, South Korea

**Keywords:** TiO2, superhydrophilic, precursor temperature, anatase phase, growth behavior

## Abstract

The hydrophilic TiO_2 _films were successfully deposited on slide glass substrates using titanium tetraisopropoxide as a single precursor without carriers or bubbling gases by a metal-organic chemical vapor deposition method. The TiO_2 _films were employed by scanning electron microscopy, Fourier transform infrared spectrometry, UV-Visible [UV-Vis] spectroscopy, X-ray diffraction, contact angle measurement, and atomic force microscopy. The temperature of the substrate was 500°C, and the temperatures of the precursor were kept at 75°C (sample A) and 60°C (sample B) during the TiO_2 _film growth. The TiO_2 _films were characterized by contact angle measurement and UV-Vis spectroscopy. Sample B has a very low contact angle of almost zero due to a superhydrophilic TiO_2 _surface, and transmittance is 76.85% at the range of 400 to 700 nm, so this condition is very optimal for hydrophilic TiO_2 _film deposition. However, when the temperature of the precursor is lower than 50°C or higher than 75°C, TiO_2 _could not be deposited on the substrate and a cloudy TiO_2 _film was formed due to the increase of surface roughness, respectively.

## Introduction

Since a TiO_2 _film showing a high refractive index is transparent in the visible light range, it can be used as an antireflection coating on a SiO_2 _thin film [1]. It can also act as a photocatalyst because of its chemical stability, high quantum yield, and nontoxic property [2]. For all of these optical applications, it is necessary to control polymorphs of TiO_2_, which have different structural and optical properties. It is well known that TiO_2 _exists in three different polymorphs: rutile, anatase, and brookite [2]. To our knowledge, brookite is an orthorhombic structure and has not been observed in thin films. Both rutile and anatase phase are tetragonal structures. The anatase phase is a low-temperature polymorph with a less dense structure (3.894 g/cm^3^) [2], an optical bandgap of 3.25 eV [3-5], and a refractive index of 2.5 [6]. TiO_2 _has been attracting much interest due to a wide range of applications such as in dye-sensitized solar cells [7,8], photocatalysts [9-12], optical coatings [6], and capacitors for large-scale integrated devices [13]. TiO_2 _photocatalysts have been applied in various fields, in which the anti-fogging, self-cleaning, or automobile windows should be quite attractive. The photocatalytic activities of TiO_2 _materials strongly depend on surface morphology, crystal structure, and crystallization of the concerned TiO_2 _photocatalyst. Various deposition techniques have been developed for depositing TiO_2 _thin films, including evaporation [3], sputtering [14], thermal oxidation of titanium [4], and the chemical vapor deposition [CVD] method [15]. Among them, the CVD technique using a metal-organic compound as a precursor [MOCVD] has many advantages, such as a good conformal coverage, the possibility of epitaxial growth and selective deposition, and the application to large-area deposition. Also, this method is of low cost, and it is easy to control the deposition growth parameters. Thus, the MOCVD method is well known as one of the most powerful techniques and is suitable for stoichiometric and microstructural thin film deposition [16].

In this experiment, therefore, we deposited TiO_2 _thin films on glass substrates with a single molecular precursor as titanium tetraisopropoxide at different precursor temperatures such as 75°C (sample A) and 60°C (sample B) by the MOCVD method. Also, we discuss the influence of the precursor temperature on the surface energy of TiO_2 _thin films.

## Experimental details

TiO_2 _thin films were deposited on a glass substrate using a MOCVD reactor, whose system was fabricated using a quartz tube and stainless steel bodies connected through O-ring joints. The MOCVD apparatus was evacuated using a rotary pump. The glass substrate was pretreated with acetone, ethanol, and deionized water in an ultrasonic cleaner and mounted onto the graphite holder that was laid in the center of the MOCVD chamber. To fix the glass substrate onto the graphite holder tightly, we grooved the graphite holder and tilted it at an angle to get a thin film with a uniform surface. The graphite holder was heated using a DC power through a super-Kanthal wire (Sandvik Korea Ltd., Seoul, South Korea) inserted in it with a substrate temperature at 400°C. The general deposition conditions are a temperature of 400°C, a working pressure of 8.2 × 10^-2 ^Torr, and a working time of 30 min. Titanium tetraisopropoxide (Ti[OCH(CH_3_)_2_]_4_) [TTIP] was used as a precursor with heating at 60°C and 75°C and without a bubbler gas. The as-grown films were characterized with X-ray diffraction [XRD], scanning electron microscopy [SEM], atomic force microscopy [AFM], Fourier transform infrared spectrometry [FT-IR], contact angle measurement, and ultraviolet-visible [UV-Vis] spectroscopy.

## Results and discussion

**Figure 1:**
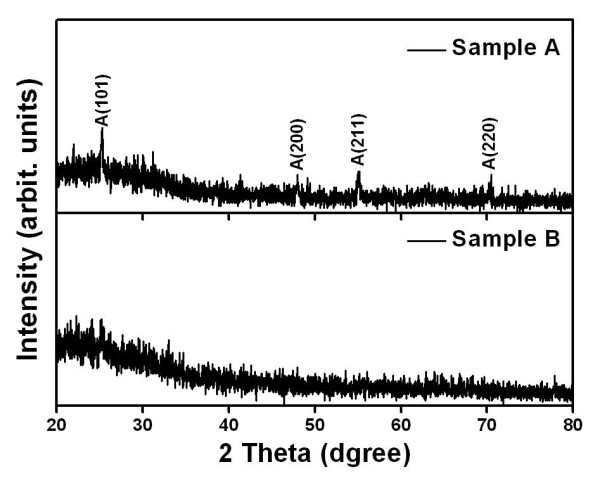
**XRD patterns of the TiO_2 _thin films at different precursor temperatures**. Sample A at 75°C and sample B at 60°C.

The structures of TiO_2 _thin films were characterized by XRD using Cu Kα radiation at 30 kV and 40 mA. Figure 1 shows the XRD patterns of the TiO_2 _thin films as a function of the precursor temperature. All the TiO_2 _thin films were deposited on the glass substrate at 400°C. Sample A showed the anatase phase and the randomly oriented polycrystalline structure. Several peaks are observed for sample A thin films at a precursor temperature of 75°C such as (101), (200), (211), and (220). On the other hand, there is no peak for sample B thin films at a precursor temperature of 60°C, indicating that the thin film is amorphous. It has been reported that the onset temperature of the thermally activated transformation from an amorphous to an anatase phase was dependent on experimental parameters such as deposition methods, deposition temperature, and different substrates. In this present work, the amorphous phase of the TiO_2 _thin films on the glass substrate at 400°C exists up to the precursor temperature of 60°C. The anatase phase appears at a precursor temperature of 75°C.

**Figure 2:**
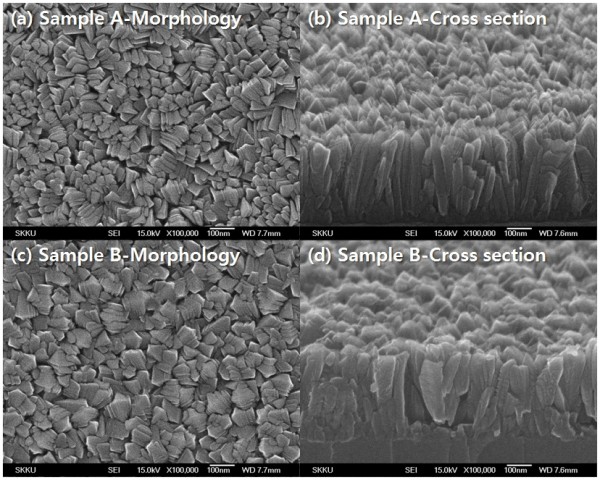
**SEM images of the TiO2 thin films at different precursor temperatures**. (**a**) Morphology and (**b**) cross section of sample A at 75°C and (**c**) morphology and (**d**) cross section of sample B at 60°C.

The influence of the precursor temperature is evidenced in the case of deposition on the glass in Figure 2a, b, c, d. The surface is composed of grains, the size of which decreases with the precursor temperature: it is about 50 nm for a growth at 75°C. In the case of a growth at a precursor temperature of 60°C, clusters of about 100 nm appear, resulting in a rougher surface. The SEM images show that the TiO_2 _grown layers have a columnar shape. The columnar structure can be evidenced by SEM images taken on cut edges perpendicular to the growth direction. Sample A has a denser surface than sample B. This phenomenon can be explained by precursor nucleation, above the substrate, the clusters being then adsorbed on the surface.

**Figure 3:**
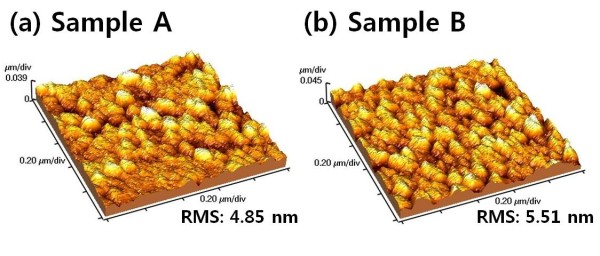
**AFM images of the TiO_2 _thin films at different precursor temperatures**. (**a**) Sample A at 75°C and (**b**) sample B at 60°C.

Figure 3 shows AFM images of the TiO_2 _thin films with different precursor temperatures such as 75°C (sample A) and 60°C (sample B). Sample A showed the flattest surface among these films. On the other hand, sample B exhibited more rough surfaces than sample A. The surface roughness values obtained by AFM for these films were 4.85 and 5.51 nm for A and B, respectively. The thickness of these films was about 300 nm. The slight change of surface roughness might result from two factors such as the limited surface diffusion caused by relatively low thermal energy and a crystallite size effect; nevertheless, our samples have similar surface roughness. This means that a change of the precursor temperature does not influence the surface roughness of TiO_2 _thin films.

**Figure 4:**
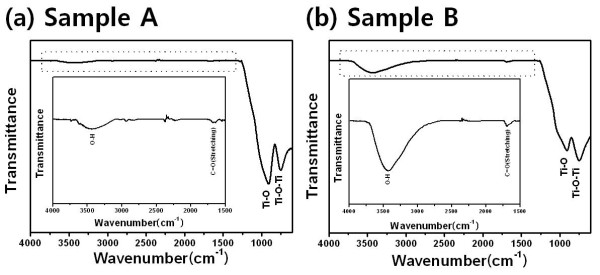
**FT-IR spectra of the TiO_2 _thin films at different precursor temperatures**. (**a**) Sample A at 75°C and (**b**) sample B at 60°C.

FT-IR spectra of the TiO_2 _thin films in Figure 4 show that peaks corresponding to stretching vibrations of O-H and C = O are around 3,300 to 3,500 and 1,600 to 1,700 cm^-1^, respectively. Also, the peak around 500 to 1,000 cm^-1 ^should be assigned to the stretching vibration of Ti-O and Ti-O-Ti. In the case of sample B, the intensity of the O-H stretching peak was increasing more than that of sample A. This means that sample B has more high surface energy than sample A.

**Figure 5:**
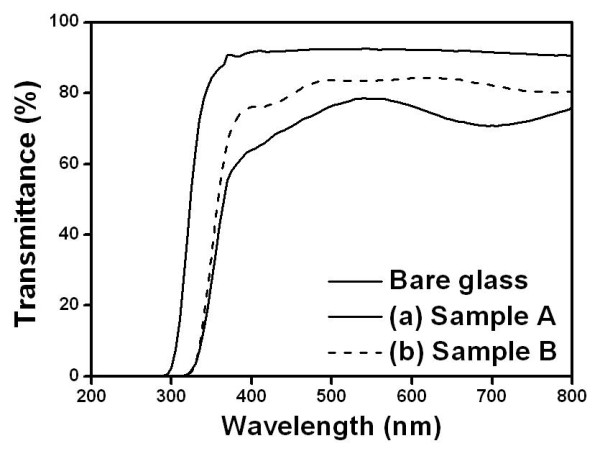
**UV-Vis spectra of the TiO_2 _thin films at different precursor temperatures**. (a) Sample A at 75°C and (b) sample B at 60°C.

Figure 5 shows the UV-Vis spectrum of deposited TiO_2 _thin films at different precursor temperatures such as 75°C (sample A) and 60°C (sample B). The TiO_2 _thin film was fast deposited at the sample A condition (precursor temperature at 75°C). This condition appeared as a haze effect and it has low transmittance. On the other case (sample B; precursor temperature at 60°C), the TiO_2 _thin film had a transmittance of about 70%.

**Figure 6:**
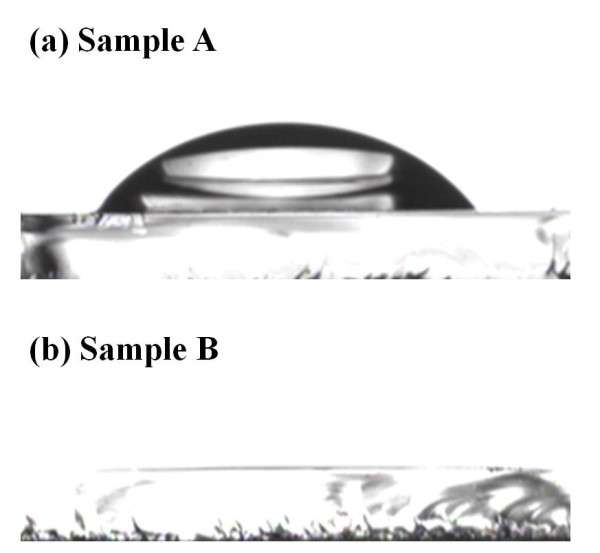
**Water contact angle images of the TiO_2 _thin films at different precursor temperatures**. (**a**) Sample A at 75°C and (**b**) sample B at 60°C.

Figure 6a, b shows the water contact angle with a change from being hydrophobic to being superhydrophilic at a precursor temperature of 75°C to 60°C. Sample A surface was observed to be hydrophobic with a contact angle of 46°. On the other hand, sample B surface was superhydrophilic between 0° and 5° at a 60°C heating treatment. There are various reasons of the change in the water contact angle, but we are convinced that the primary factor of the precursor heating treatment is the surface functional group. Therefore, SEM and XRD were employed for the confirmation of changed roughness, and FT-IR analysis was performed for the verification of changed O-H surface functional group. At the precursor temperature of 60°C, the thin film was grown in the amorphous phase and has an increasing coordination number. This means that it has a surface energy which is higher than that of the crystalline thin film. Therefore, sample B has a superhydrophilic surface.

## Conclusions

The hydrophilic TiO_2 _films were successfully deposited on slide glass substrates using TTIP as a single precursor without carriers or bubbling gases by the MOCVD method. The amorphous phase of the TiO_2 _thin films on the glass substrate at 400°C exists up to the precursor temperature of 60°C. The anatase phase appears at a precursor temperature of 75°C. The columnar structure can be evidenced by SEM images taken on cut edges perpendicular to the growth direction. Sample A has a denser surface than sample B. This phenomenon can be explained by precursor nucleation, above the substrate, the clusters being then adsorbed on the surface. XRD data show that the phase was diverted from amorphous to anatase, and FT-IR analysis was performed for the verification of changed O-H surface functional group. At the precursor temperature of 60°C, the thin film was grown in the amorphous phase and has an increasing coordination number.

## Competing interests

The authors declare that they have no competing interests.

## Authors' contributions

S-HN and J-HB conceived the study. S-HN carried out the experiments. S-JC contributed the analysis of the study. S-HN drafted the manuscript. All authors are involved in revising the manuscript and approved the final version.
